# Prediction of subsolid pulmonary nodule growth rate using radiomics

**DOI:** 10.1186/s12880-023-01143-x

**Published:** 2023-11-07

**Authors:** Zong Jing Ma, Zhuang Xuan Ma, Ying Li Sun, De Chun Li, Liang Jin, Pan Gao, Cheng Li, Ming Li

**Affiliations:** Department of Radiology, Huadong Hospital, Fudan University, Shanghai, 200040 China

**Keywords:** Computed tomography, Growth, Radiomics, Subsolid pulmonary nodules

## Abstract

**Background:**

Pulmonary nodule growth rate assessment is critical in the management of subsolid pulmonary nodules (SSNs) during clinical follow-up. The present study aimed to develop a model to predict the growth rate of SSNs.

**Methods:**

A total of 273 growing SSNs with clinical information and 857 computed tomography (CT) scans were retrospectively analyzed. The images were randomly divided into training and validation sets. All images were categorized into fast-growth (volume doubling time (VDT) ≤ 400 days) and slow-growth (VDT > 400 days) groups. Models for predicting the growth rate of SSNs were developed using radiomics and clinical features. The models’ performance was evaluated using the area under the curve (AUC) values for the receiver operating characteristic curve.

**Results:**

The fast- and slow-growth groups included 108 and 749 scans, respectively, and 10 radiomics features and three radiographic features (nodule density, presence of spiculation, and presence of vascular changes) were selected to predict the growth rate of SSNs. The nomogram integrating radiomics and radiographic features (AUC = 0.928 and AUC = 0.905, respectively) performed better than the radiographic (AUC = 0.668 and AUC = 0.689, respectively) and radiomics (AUC = 0.888 and AUC = 0.816, respectively) models alone in both the training and validation sets.

**Conclusion:**

The nomogram model developed by combining radiomics with radiographic features can predict the growth rate of SSNs more accurately than traditional radiographic models. It can also optimize clinical treatment decisions for patients with SSNs and improve their long-term management.

**Supplementary Information:**

The online version contains supplementary material available at 10.1186/s12880-023-01143-x.

## Introduction

Lung cancer is the malignant tumor with the highest incidence and mortality rate in the world, and its incidence rate is rapidly increasing in China, ranking first among malignant tumors [1]. Early detection, diagnosis and treatment are the most effective methods to prolong the survival of lung cancer patients. The low-dose computed tomography (LDCT) screening can detect stage I lung cancer and significantly reduce mortality in high-risk screeners, which has become a worldwide reliable method for lung cancer screening in high-risk populations [2], however, with the widespread use of LDCT and the continuous development of software for identifying pulmonary nodules, a large number of isolated pulmonary nodules have been detected, particularly for sub-solid pulmonary nodules(SSNs) [3, 4]. SSNs include ground-glass nodules (GGNs) and part-solid nodules (PSNs) [5], compared to solid nodules (SNs), the clinical course of SSNs is more slowly and have a significantly improved prognosis if treated immediately [6, 7].

Accurate identification of benign and malignant pulmonary nodules is a critical issue in lung cancer prevention and treatment, and the development of a standardized follow-up strategy for the evaluation of pulmonary nodules can help improve the effectiveness of diagnosis and treatment. However, some nodules are difficult to evaluate because they lack distinguishing radiographic characteristics or are small in size and CT follow-up is often needed to monitor their dynamic changes. Current protocols for follow-up of pulmonary nodules are sketchy, and the duration of follow-up is broad and generalized; Hammer et al. [8] showed that the growth rate of pulmonary nodules is closely related to the benign or malignant nature of the nodules, and that faster-growing nodules have a greater likelihood of developing into malignant nodules in the future, but they still need to be differentiated from inflammatory nodules. Therefore, an accurate understanding of the growth characteristics and growth trends of pulmonary nodules is essential for optimizing follow-up protocols. The assessment of pulmonary nodule growth includes the measurement of two-dimensional diameter, three-dimensional volume, changes in density, volume doubling time (VDT), and mass doubling time (MDT). Previous studies have revealed many factors associated with pulmonary nodule growth, such as nodule size [9, 10], density [11, 12], morphology (lobulation [12], bubble [13], and air-bronchogram sign [9]), surrounding structures [12] (pleural attachment, vascular change) and clinical history (advanced age [9], history of smoking [12, 14], and history of lung cancer [9, 15, 16]). However, assessment of these signs is influenced by the level of the evaluator's imaging and it requires more time to assess. Radiomics can extract a large number of features from images and transform them into comprehensive quantitative data via high-throughput computing [17]. Previous studies have demonstrated the clinical value of CT-based radiomics in distinguishing between benign and malignant lung lesions [18, 19], identifying the aggressiveness of lung adenocarcinoma [20, 21], and predicting distant and lymph node metastases [22, 23]. Fewer studies have been performed on the growth rate of lung nodules. Therefore, the aim of the present study was to utilize clinical and radiomics features to develop models for predicting the growth rate of SSNs to minimize radiation exposure and healthcare costs, and to provide timely and effective clinical interventions for patients with early-stage lung cancer.

## Materials and methods

### Ethics statements

This retrospective study was approved by our institutional review boards (number: NO.2019K134). This study was approved by Huadong Hospital Affiliated Fudan University institutional ethics committee and the requirement for informed consent was waived. The procedures were all carried out in line with the relevant guidelines and regulations.

### Study design and data collection

Two radiologists (S. Y. L., seven years of experience in thoracic radiology; M. Z. X., three years of experience in thoracic radiology) searched picture archiving and communication systems in Huadong Hospital affiliated to Fudan University for the following keywords: subsolid nodules, ground-glass nodules, ground glass opacity, part-solid nodules and mixed ground glass nodule. Then, the growing SSNs were identified from the above cases, and the criteria for growth were defined by the Fleischner Society 2017 guidelines as an increase of 2 mm in nodule diameter on two CT images [24]. Cases with inconsistent results were re-evaluated by a radiologist (L. M., with >20 years of experience in thoracic radiology). The inclusion criteria were as follows: (a) SSNs with a diameter of <3 cm and (b) at least two thin-section CT images with a time interval greater than 30 days. The exclusion criteria were as follows: (a) poor image quality due to respiratory or metallic artifacts and (b) images with reduced diameter compared to previous CT scans.

**Fig. 1:**
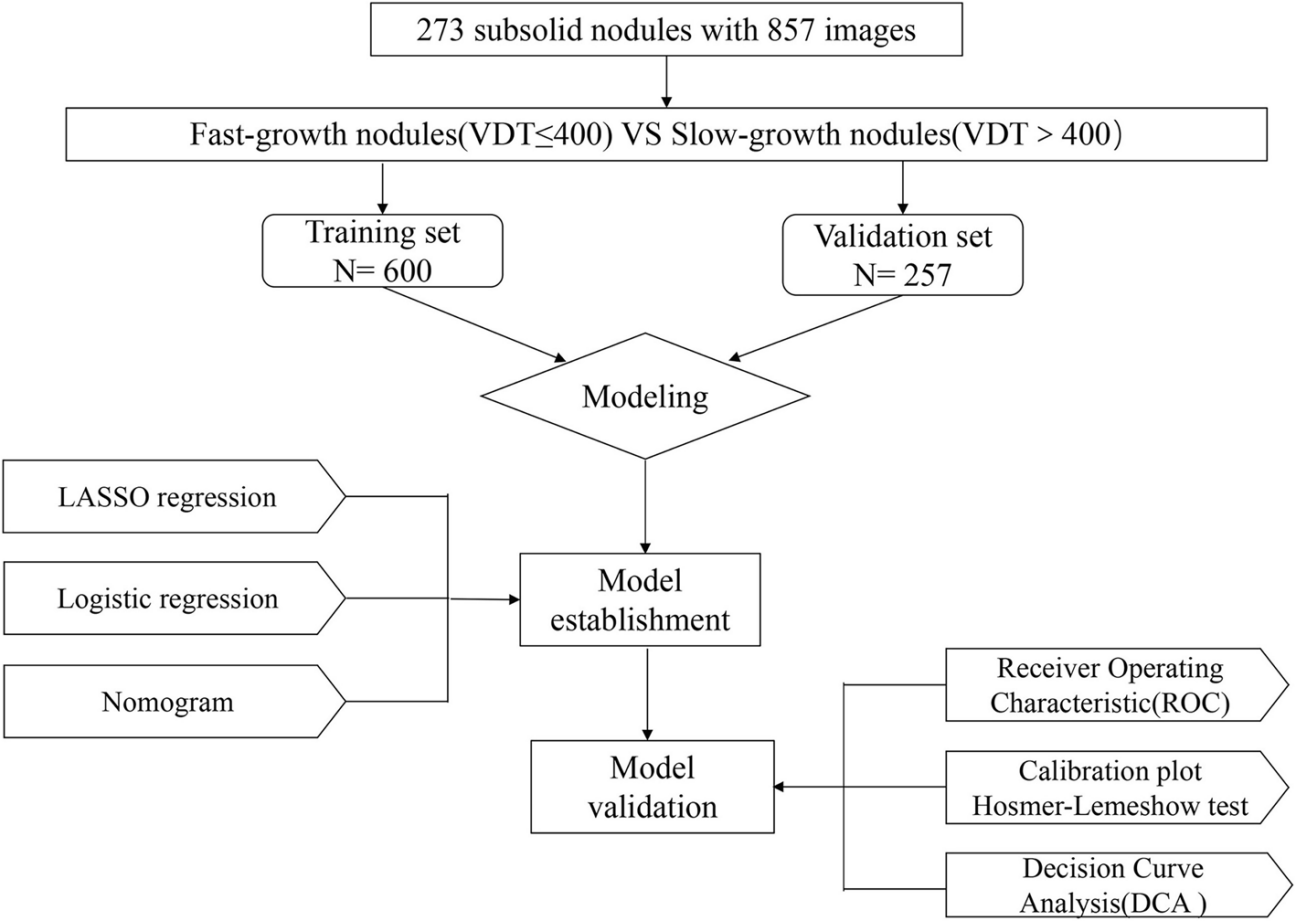
Study workflow diagram. VDT = volume doubling time, LASSO = least absolute shrinkage and selection operator, ROC = receiver operating characteristic curve, DCA = decision curve analysis

**Fig. 2:**
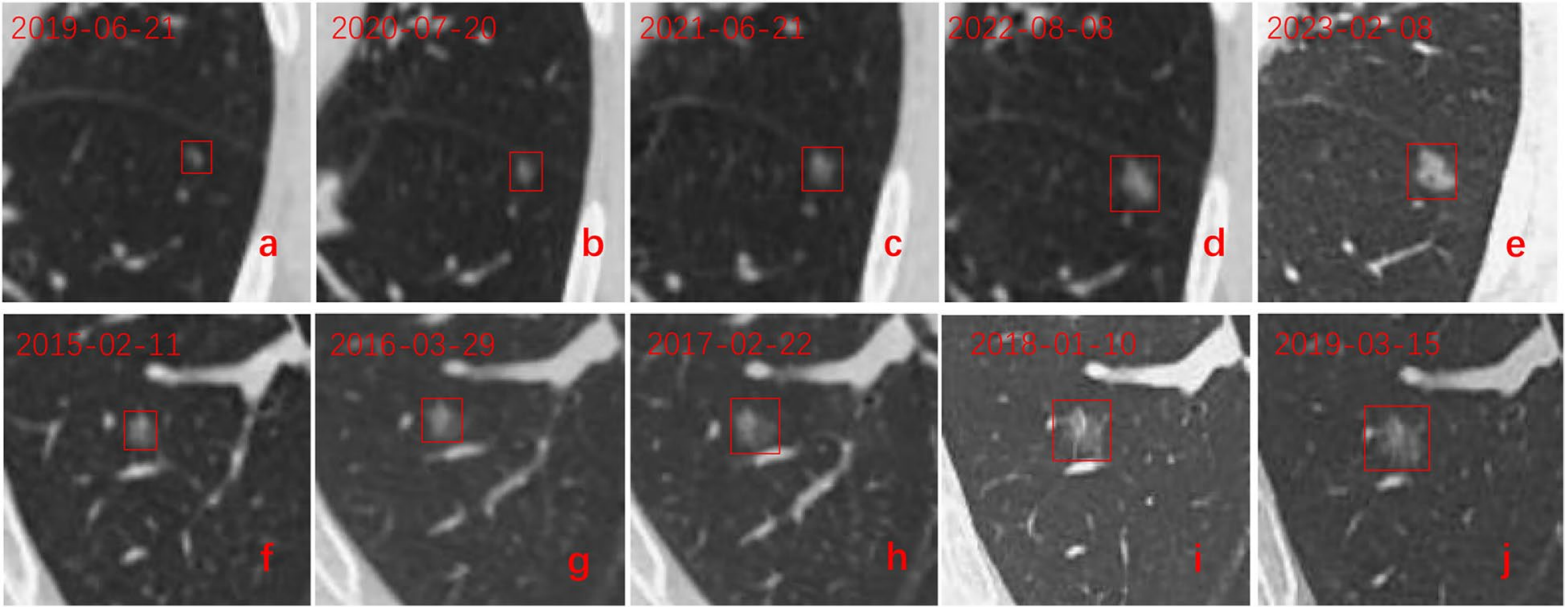
Two patients with GGNs. **a**–**e** A female with a GGN in the left lower lobe. The GGN was incidentally discovered on June 21, 2019. At the follow-up, the VDT was fewer than 400 days and was classified into the fast-growth group. **f**–**j** A male with a GGN in the right lower lobe. The GGN was incidentally discovered on February 11, 2015. At the follow-up, the VDT was more than 400 days and was classified into the slow-growth group. GGNs: ground glass nodules, VDT = volume doubling time

A total of 273 SSNs (mean age: 63.22 years, 26–96 years) were included in the final analysis for a total of 857 thin-section CT images. Each image was included as a separate case in the study and randomly assigned into the training and validation sets at a 7:3 ratio. The flow chart for the study is shown in Fig. 1. Two adjacent patient images were used to calculate the VDT. The last image was used to calculate the VDT of the nodule in the second to last image, which was not included as a separate case (Fig. 2). When a patient had multiple growing pulmonary nodules, we select the most suspicious PSN based on the following rules [25]: (a)PSNs had priority over GGNs, (b)when there were≥2 GGNs, the largest GGN was selected, and (c)in the case of ≥2PSNs, the PSN with the largest solid part was selected. The mean interval between CT images was 439.74 days (range 33–3,142 days) with a median of 358 days.

### CT examination

All CT scans were performed using one of the following four scanners: GE Discovery CT750 HD (GE Healthcare, USA), 64-slice Light Speed VCT (GE Healthcare, USA); Somatom Definition Flash (Siemens Healthcare, Germany) and Somatom Sensation-16 (Siemens Healthcare, Germany). The scan parameters were as follows: tube current was 120–200 mA; tube voltage was 80–120 kV; scan layer thickness was 1–2 mm; reconstruction algorithm was STND/medium sharp; scan phase was deep inspiratory phase; and scan body position was supine.

### Radiographic feature assessment and clinical information collection

Two radiologists (J. L., six years of experience in thoracic radiology; L. D. C., two years of experience in thoracic radiology) who were blinded to the growth rate of SSNs evaluated and recorded the radiographic features of each CT examination. Disagreements were resolved with a discussion. The evaluation conditions were a window width of 1,500 Hounsfield units [HU] and a window level of -700 HU. The following radiographic information was recorded: location (upper, middle, and lower lobe of the right lung, upper and lower lobe of the left lung), density (GGNs, PSNs), shape (round, oval, irregular), lobulation (yes, no), spiculation (yes, no), bubble (yes, no), vascular changes (yes, no), bronchiole change (yes, no), and pleural attachment (yes, no). Clinical information was collected and recorded by M. Z. J. (four years of experience in thoracic radiology), including age, sex, previous history of lung cancer, and whether pathological results were obtained.

### Nodule segmentation and VDT acquisition

Medical image processing and navigation software 3D Slicer ((version 4.8.0, Brigham and Women's Hospital, https://www.slicer.org/) was used to manually delineate the volume of interest (VOI) by a radiologist (M. Z. J.). Then, the VOI was confirmed by another radiologist (G.P., six years of experience in thoracic radiology). The surrounding vascular, bronchiole, and pleural structures were avoided during the delineation process. Finally, the VOI data for all SSNs were preserved in the form of NII (desensitization format) for subsequent analysis.

VDT is a significant indicator used to quantify the growth rate of lung nodules [26]. Most studies [27–29] defined pulmonary nodules with a VDT > 400 days and VDT ≤ 400 days as slow growing and fast-growing pulmonary nodules, respectively. The same criteria were adopted for the present study. Images other than the last image were included in the study as separate cases. The following formula was used to calculate VDT: $VDT=\left(volume\;doubling\;time\right)=\frac{\left(T1-T0\ast\log2\right)}{log\left(V1/V0\right)}$, where V0 and V1 represent the volumes at T1 (time 1—the next examination date) and T0 (time 0—the first examination date).

### Feature extraction and repeatability analysis of radiomics

The Pyradiomics Toolkit, version 2.1.0 (https://github.com/AIM-Harvard/pyradiomics) was used to extract 1,218 radiomics features from each lesion. To investigate the inter- and intra-observer reproducibility of the radiomics feature extraction, 60 images were randomly selected from the training set one month after outlining is completed and the VOIs of the nodules were outlined by two radiologists (M. Z. J. and L. D. C.). Two-way random effects models were used to calculate intraclass correlation coefficients (ICCs) to determine inter- and intra-observer reliabilities. Only the radiomics features with excellent reliability (ICC ≥ 0.80) were considered robust.

### Feature selection, model development, and validation

The performance of a predictive model depends on the amount of useful information, and in order to remove irrelevant features to enhance the stability of the model and to prevent overfitting, Clinical and radiological characteristics of the fast- and slow-growth groups were compared using one-way analysis of variance (ANOVA). Then, binary logistic regression test was used for the multivariate analysis to develop the clinical model.

First, in the training set, ANOVA was used to identify potentially significant radiomics features (*p* < 0.05). Then, the least absolute shrinkage and selection operator (LASSO) method with penalty tuning conducted using 10-fold cross validation was used to select the most stable radiomics feature to form a radiomics signature.. Multivariate logistic regression analysis using backward stepwise selection was performed to develop the model by incorporating the radiomics signature and clinical features. An individualized prediction nomogram was constructed based on the results of the multivariate logistic regression analysis.

Each model’s performance in distinguishing the fast- and slow-growth groups was analyzed based on the area under the curve (AUC) values for the receiver operating characteristic (ROC) curves in the training and validation sets. The ROC curves between different models were compared using the DeLong’s test [30]. The calibration curves plotted using the Hosmer–Lemeshow test were applied to assess the goodness of fit of the radiomics nomogram predictions and observation outcomes in the primary and validation groups. Decision curve analysis was conducted to determine the clinical usefulness of the nomogram.

### Statistical analysis

All statistical analyses were performed using R statistical software, version 4.1.0 (https://www.r-project.org/) and a commercially available software program SPSS 23.0 for Windows (SPSS, Chicago, IL, USA). Continuous variables were expressed as means ± standard deviations and compared using independent samples t-test or Kruskal–Wallis test. Categorical variables were expressed using frequency and analyzed via chi-square or Fisher’s exact test. The AUC for the ROC curve was used to evaluate the predictive effectiveness of the model. DeLong's test was applied to test the statistical significance of the AUC values for different models. Calibration curves were plotted using the Hosmer–Lemeshow test to assess the fitness of the nomogram predictions. Clinical decision curves were utilized to evaluate the clinical usefulness of the models. A two-sided *p*-value of < 0.05 was considered statistically significant.

## Results

### Comparison of radiographic features between fast- and slow-growth SSNs

A total of 273 SSNs with 857 CT scans were analyzed (112 men, 161 women; mean age: 63.22 ± 14.09 years). Clinical and radiographic patient characteristics in the training and validation sets are presented in Table 1. There was no statistically significant difference in the distribution of the clinical and radiographic features between the training and validation sets.

**Table 1:** Patient information for the training and validation sets

Characteristic		Training cohort	Validation cohort	*P* value
Participants		600	257	
Age(year)		62.91 ± 14.11	63.93 ± 14.05	0.332
Gender				0.397
	Male	275(45.83%)	120(46.70%)	0.817
	Female	325(54.17%)	137(53.31%)	
Density				0.995
	GGN	238(39.67%)	102(39.69%)	
	PSN	362(60.33%)	155(60.31%)	
Shape				0.912
	Round	157(26.17%)	70(27.24%)	
	Oval	51(8.5%)	23(8.95%)	
	Irregular	392(65.33%)	164(63.81%)	
Location				0.862
	RU	223(37.17%)	96(37.35%)	
	RM	61(10.17%)	24(9.34%)	
	RL	94(15.67%)	34(13.23%)	
	LU	150(25.00%)	70(27.24%)	
	LL	72(12.00%)	33(12.84%)	
Lobulation	Present	446(74.33%)	188(73.15%)	0.718
	Absent	154(25.67%)	69(26.84%)	
Spiculation	Present	328(54.67%)	142(55.25%)	0.874
	Absent	272(45.33%)	115(44.75%)	
Bubble	Present	60(10.00%)	35(13.62%)	0.218
	Absent	540(90.00%)	222(86.38%)	
Bronchiole change	Present	211(35.17%)	98(38.13%)	0.407
	Absent	389(64.83%)	159(61.87%)	
Vascular change	Present	129(21.50%)	51(19.84%)	0.586
	Absent	471(78.50%)	206(80.16%)	
Pleural attachment	Present	187(31.17%)	85(33.07%)	0.583
	Absent	413(68.83%)	172(66.93%)	
History of lung cancer	Present	226(37.67%)	101(39.30%)	0.652
	Absent	374(62.33%)	156(60.70%)	
Growth rate	Fast-growth nodules	71(11.83%)	37(14.40%)	0.3
	Slow-growth nodules	529(88.17%)	220(85.60%)	

Age is represented as the mean ± standard deviation; other data are represented as the number of patients with the percentage in parentheses

*PSN* part-solid nodule, *GGN* ground-glass nodule, *RU* right upper, *RM* right middle, *RL* right lower, *LU* left upper, *LL* left lower

### Feature selection and establishment of the model

Univariate analysis revealed that nodule density, presence of spiculation, presence of vascular changes, and history of lung cancer were significantly different between the fast- and slow-growth SSNs in the training set (all *p* < 0.05; Table 2). Binary logistic regression analysis showed that nodule density, presence of spiculation, and presence of vascular changes were independent predictors of the SSN growth rates (all *p* < 0.05; Table 3). The above features were used to develop radiographic models.

**Table 2:** Patient information for the training sets

Characteristic		Fast-growth	Slow-growth	*P* value
Participants		71	529	
Age (year)		62.85 ± 12.01	62.78 ± 14.36	0.552
Gender				0.533
	Male	35(49.30%)	240(45.37%)	
	Female	36(50.70%)	289(54.63%)	
Density				
	GGN	43(60.56%)	195(36.86%)	< 0.001*
	PSN	28(39.44%)	334(63.14%)	
Shape				0.312
	Round	15(21.13%)	142(26.84%)	
	Oval	4(5.63%)	47(8.88%)	
	Irregular	52(73.24%)	340(64.27%)	
Location				0.764
	RU	28(39.44%)	195(36.86%)	
	RM	9(12.68%)	52(9.83%)	
	RL	9(12.68%)	85(16.07%)	
	LU	15(21.13%)	135(25.52%)	
	LL	10(14.04%)	62(11.72%)	
Lobulation	Present	55(77.46%)	391(73.91%)	0.52
	Absent	16(22.54%)	154(26.09%)	
Spiculation	Present	55(77.46%)	273(51.61%)	< 0.001*
	Absent	16(22.53%)	256(48.39%)	
Bubble	Present	11(15.49%)	49(9.26%)	0.22
	Absent	60(84.51%)	480(90.74%)	
Bronchiole change	Present	27(38.03%)	184(34.78%)	0.591
	Absent	44(61.97%)	345(65.22%)	
Vascular change	Present	23(32.39%)	106(20.04%)	0.017*
	Absent	48(67.60%)	423(79.96%)	
Pleural attachment	Present	24(33.80%)	163(30.81%)	0.61
	Absent	47(66.20%)	366(69.19%)	
History of lung cancer	Present	40(56.34%)	186(35.16%)	< 0.001*
	Absent	31(43.66%)	343(64.84%)	

Age is represented as the mean ± standard deviation; other data are represented as the number of patients with the percentage in parentheses

*PSN* part-solid nodule, *GGN* ground-glass nodule, *RU* right upper, *RM* right middle, *RL* right lower, *LU* left upper, *LL* left lower

^*^p-value < 0.05

**Table 3:** Multivariate analysis of clinical and radiographic features

Characteristic	OR (95% CI)	*P*
Density	1.964 (1.148–3.360)	0.014*
Spiculation	2.557 (1.329–4.921)	0.005*
Vascular change	1.954 (1.116–3.422)	0.019*
History of lung cancer	1.294 (0.725–2.307)	0.383

*OR* odds ratio, *CI* confidence interval

^*^p-value < 0.05

A total of 60 SSNs were randomly selected from the training set and delineated again by M. Z. J. and L. D. C. one month after the initial analysis. The results of the inter- and intra-observer Bland-Altman plots showed that there was a high level of agreement between observers M. Z. J. and L. D. C. The intra-observer consistency was higher than the inter-observer consistency. Additional details about the ICC analysis can be found in the Supplemental data.

**Fig. 3:**
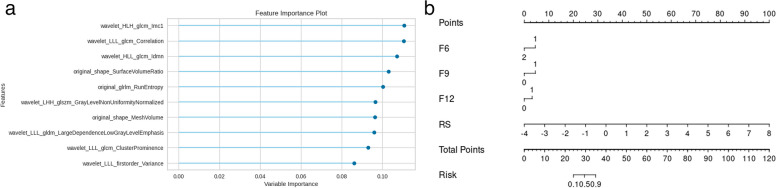
The selected radiomics features and the final nomogram. **a** The 10 selected features and their corresponding coefficients. **b** The nomogram was developed incorporating a radiomics signature with radiographic features. F6 = nodule density, 1 = ground-glass nodule, 2 = part-solid nodule, F9 = spiculation, F12 = vascular change

Among 1,218 features, 788 robust radiomics features (ICC ≥ 0.8) were selected for further analysis. A total of 221 features with statistically significant differences were selected using ANOVA (*p* < 0.05). Then, LASSO analysis was carried out to select the optimized subset of features to construct the final model. Additional details about the LASSO analysis can be found in the Supplemental data. The most predictive subset of features was selected after determining the number of features and the corresponding coefficients were evaluated. The radiomics signature was calculated by summing the selected features weighted by their coefficients. Logistic regression analysis using backward stepwise selection identified the radiomics signature, nodule density, presence of spiculation, and presence of vascular changes as independent predictors, which were incorporated to develop an individualized prediction nomogram model (Fig. 3).

### Performance of the radiographic model, radiomics signature, and nomogram model

**Fig. 4:**
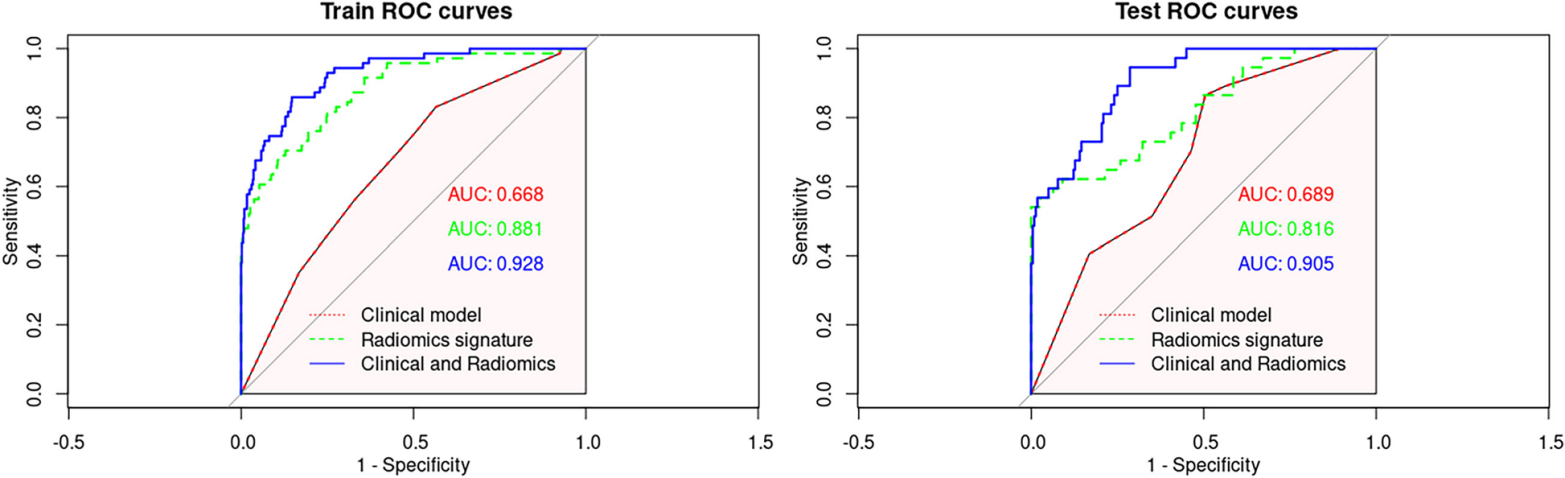
The ROC curves for the training and validation sets. ROC = receiver operating characteristic curve

The ROC curve was used to evaluate the model performance. The radiographic model, radiomics signature, and nomogram model in the training set achieved AUCs of 0.668 (95% confidence interval (CI): 0.618, 0.696), 0.881 (95%CI: 0.852, 0.906), and 0.928 (95%CI: 0.904, 0.947), respectively. The radiographic model, radiomics signature, and nomogram model in the validation set achieved AUCs of 0.689 (95%CI: 0.620, 0.738), 0.816 (95%CI: 0.774, 0.870), and 0.905 (95%CI: 0.855, 0.933), respectively. In both the training and validation sets, the nomogram performed significantly better than the radiomics and radiographic models (DeLong’s test, *p* < 0.05; Fig. 4).

### Calibration and clinical utility of models

**Fig. 5:**
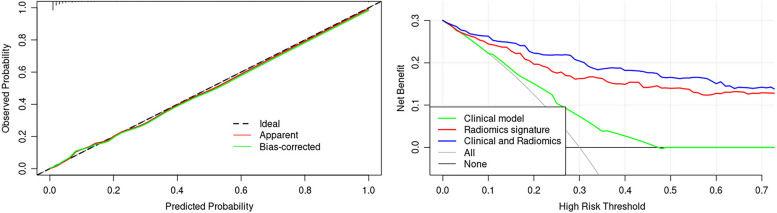
**a** Calibration curve shows that the growth rate probabilities predicted by the nomogram agreed well with the actual probabilities. **b** Decision curve analysis of the models. The nomogram model had a higher net benefit than the other two models across most threshold probabilities

The calibration curve for the nomogram showed good calibration in the training and validation sets. The decision curve suggested that in both the training and validation sets, clinical decisions based on the nomogram and radiomics had increased net benefits compared to the all-treatment and no-treatment scenarios. The nomogram performed slightly better than radiomics for most threshold probabilities (Fig. 5).

## Discussion

The present study established a nomogram model that combines radiographic features and radiomics to predict the growth rate of sub-solid nodules. It showed the best performance in the training and validation sets (AUC: 0.928, 95%CI: 0.904–0.947; 0.905, 95%CI: 0.855–0.933, respectively). The radiomics model also outperformed the radiographic model in the training and validation sets, and the addition of radiographic information did not significantly improve the radiomics model’s performance. The model could inform the likely growth pattern of the nodule before the actual follow-up scans, which can help to formulate a scientific and standardized follow-up strategy, improve the level of accurate diagnosis, and improve the effectiveness of diagnosis and treatment.

Radiomics is widely used in clinical research by mining the invisible but clinically significant information from images to reflect the spatial heterogeneity, microenvironment, and gene expression of lesions [31]. Tan et al. [32] integrated clinical and radiomics features to establish a nomogram for predicting the growth rate of early lung adenocarcinoma. Unlike the present study, their study included SSNs with pathological results, while in clinical practice, most pulmonary nodules are often observed via follow-up without surgery, thus limiting the application of the model. Furthermore, their study included only baseline images and the last CT image before surgery, and the AUC for the established nomogram in the validation set was 0.78, which may have been caused by the relatively small sample size. Apart from their study, the present investigation showed that radiomics can also predict the growth rate of subsolid nodules without pathological results. Xue et al. [33] established a nomogram predicting the growth of indeterminate small pulmonary nodules on high resolution CT over the course of two years by integrating radiomics features with information about the patient's gender and nodule type. However, this study only included nodules with pathology results, resulting in a relatively high proportion of malignant nodules. Yang et al. [34] developed several machine learning models for predicting whether a pulmonary nodule grows within one year and found that the logistic regression model combining age and radiomics features performed best (AUC of 0.87 in the training set; AUC of 0.82 in the validation set). This study also had a relatively small sample size and a third of the patients were missed during the follow-up, resulting in selection bias. The performance of the radiomics model developed in our study for predicting the growth rate of SSNs exceeded the traditional radiographic features, which further demonstrates the potential of radiomics in exploring imaging information for tumor heterogeneity. In the context of deep learning, Liao et al. [35] built a SiamModel for predicting the growth of SSNs. It outperformed the radiomics model in both the NLST validation set and the external test set, demonstrating a significant application of deep learning for growth prediction. However, there are few studies using deep learning for pulmonary nodule growth prediction, and most of them are single-center retrospective studies with small sample sizes and inconsistent acquisition protocols. Prospective multicenter studies with larger sample sizes are needed for future exploration.

Evaluation of the benign and malignant nature of a clinically detected pulmonary nodule is important, and there are many models that integrate clinical and radiomics features to predict the nature of nodules [18]. However, follow-up is required in most cases of indeterminate nodules with unremarkable imaging signs. Previous studies have found that factors associated with the growth of pulmonary nodules are mainly radiographic features, such as the size, morphology, and surrounding structures of the nodule. Similarly, in this study, we found that nodule density, spiculation, and vascular changes were associated with the nodule growth rate. Compared to GGN, PSN are denser, more aggressive and grow faster; spiculation is defined as small spiny protuberance at the edge of a nodule, indicating an aggressive lesion with infiltrative growth into the periphery, which is a sign of malignancy; vascular changes are defined as thickening and tortuosity of blood vessels crossing the lesion or aggregation of blood vessels around the lesion, suggesting that the lesion is more demanding of blood supply, and is also a sign of malignancy [5, 36]. The volume of nodules is an essential feature to determine whether nodules grow or not. In this study, the final selected radiomics features included nodule volume, indicating that it is not only a predictor of whether nodules grow or not, but also an important predictor of the growth rate of SSNs. However, the prediction model built based on imaging information alone had a low performance, which may have been due to the lack of typical imaging signs in these nodules. Moreover, the assessment of these signs is subjective and inaccurate. Therefore, determining the growth rate of SSNs based solely on the imaging features is unreliable.

This study had several limitations. First, it was a single-center study, and differences in CT scanning equipment and parameters may have affected the radiomics features. The retrospective data collection method is subject to selection bias, and a larger external data set is needed for future validation. Second, the nodule growth was defined by changes in nodule size outlined by hand, but the presence of subjective factors (inaccurate outline and errors) may have caused variability in the results. Although intra-class correlation analysis was performed to show that the study results had a high level of consistency, more rigorous analysis is needed in the future. Third, VDT in this study was used to determine the growth rate of nodules, but in clinical conditions, there are some nodules that show growth as an increase in the solid component inside the nodule without any change in the volume. In such cases, the use of MDT is superior to VDT. Fourth, the majority of nodules included in this study had no pathological results, and the short VDT of the nodules did not always mean that the nodules were malignant, since inflammatory lesions were also possible. However, the follow-up period was relatively long and excluded images of nodules that were smaller than those obtained in previous CT scans. Lastly, in this study, we used binary logistic regression to combine clinical and radiographic patient characteristics, but this approach is restricted by the limitations of the algorithm, in Chandra's [37] study, three methods for combining between different types of data were established, which has implications for the mutual fusion of clinical and radiographic patient characteristics.

## Conclusions

In conclusion, we have established a model to predict the growth rate of SSN by combining traditional radiological information and radiomics information, which is better than the radiological model and radiomics model, and can predict the growth trend of SSN more accurately. This model could help radiologists optimize the follow-up management of patients with SSN and could reduce unnecessary diagnostic interventions.

### Supplementary Information

**Additional file 1: Supplementary Figure 1.** Inter- and intra-observer Bland-Altman plots of measurement variability in 60 SSNs. SSNs=subsolid nodules, CI=confidence interval.** Supplementary Figure 2.** The process of select radiomics features. LASSO=least absolute shrinkage and selection operator.

## Data Availability

The raw datasets used during the current study are available from the corresponding author on reasonable request.

## Abbreviations

AUC: Area under the curve
CT: Computed tomography
DCA: Decision curve Analysis
GGNs: Ground glass nodules
ICC: Intraclass correlation coefficient
LASSO: Least absolute shrinkage and selection operator
LDCT: Low dose computed tomography
HRCT: High resolution computed tomography
HU: Hounsfield units
NLST: National Lunch Screening Trial
PSNs: Part-solid nodules
ROC: Receiver operating characteristic curve
SNs: Solid nodules
SSNs: Subsolid nodules
VOI: Volume of interest
VDT: Volume doubling time
