# Supplementary material for: Prediction of subsolid pulmonary nodule growth rate using radiomics
Source: BMC Med Imaging. 2023 Nov 7;23:177. doi: 10.1186/s12880-023-01143-x (PMC10629176; doi:10.1186/s12880-023-01143-x)
Supplement: Supplementary file 1 — Additional file 1: Supplementary Figure 1. Inter- and intra-observer Bland-Altman plots of measurement variability in 60 SSNs. SSNs=subsolid nodules, CI=confidence interval. Supplementary Figure 2. The process of select radiomics features. LASSO=least absolute shrinkage and selection operator. [file 12880_2023_1143_MOESM1_ESM.zip › clean version-Supplementary data.docx]

**Supplementary data**

Supplementary Figure 1 shows the inter- and intra-observer Bland-Altman plots for 60 SSNs. The 95% CIs for inter- and intra-observer variability were -0.27, 0.25 and -0.20, 0.20, respectively. This indicates that there was a high consistency between observers M and L and that the intra-observer consistency was higher than the inter-observer consistency.

The process to select radiomics features is shown in Supplementary Figure 2. Variable selection using LASSO binary logistic regression model. A coefficient profile plot was constructed against the log(lambda) sequence.

**Supplementary Figure Legends**

**Supplementary Figure 1.** Inter- and intra-observer Bland-Altman plots of measurement variability in 60 SSNs. SSNs=subsolid nodules, CI=confidence interval.

**Supplementary Figure 2.** The process of select radiomics features. LASSO=least absolute shrinkage and selection operator.
