# Supplementary figures and images for: Prediction of subsolid pulmonary nodule growth rate using radiomics
Source: BMC Med Imaging. 2023 Nov 7;23:177. doi: 10.1186/s12880-023-01143-x (PMC10629176; doi:10.1186/s12880-023-01143-x)

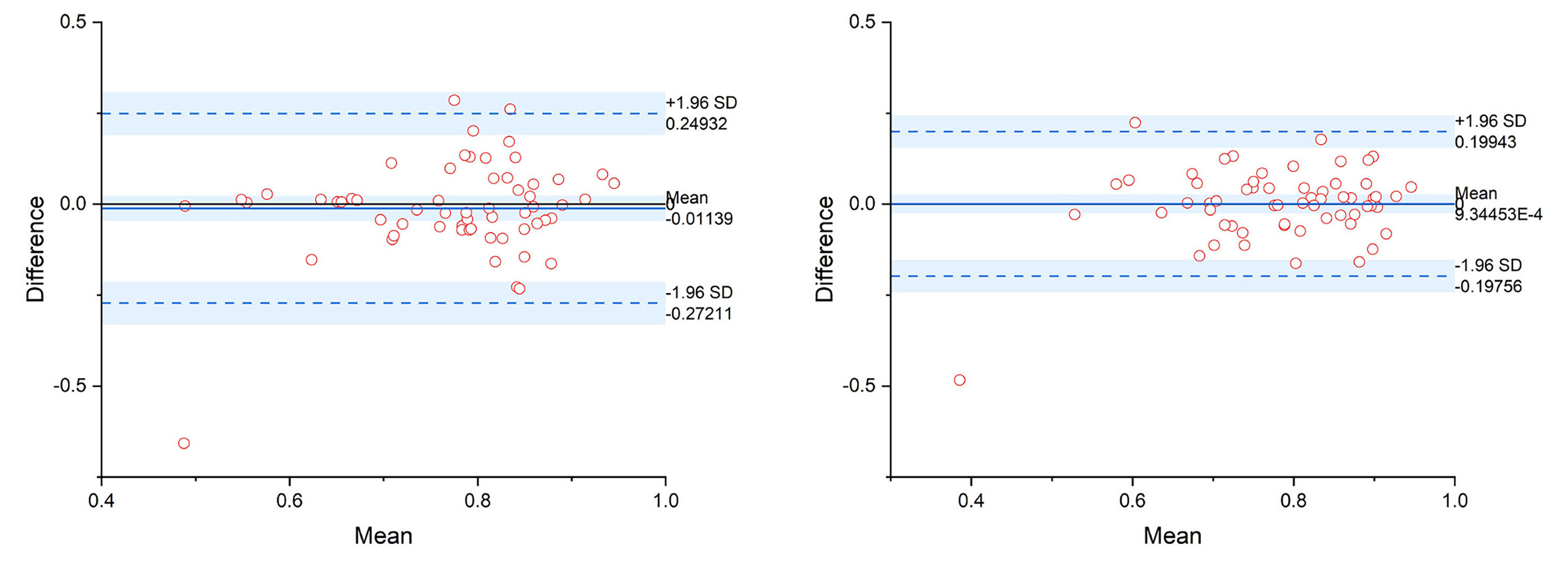

Supplement: Supplementary file 1 — Additional file 1: Supplementary Figure 1. Inter- and intra-observer Bland-Altman plots of measurement variability in 60 SSNs. SSNs=subsolid nodules, CI=confidence interval. Supplementary Figure 2. The process of select radiomics features. LASSO=least absolute shrinkage and selection operator. [file 12880_2023_1143_MOESM1_ESM.zip › Supplementary Figure 1.jpg]

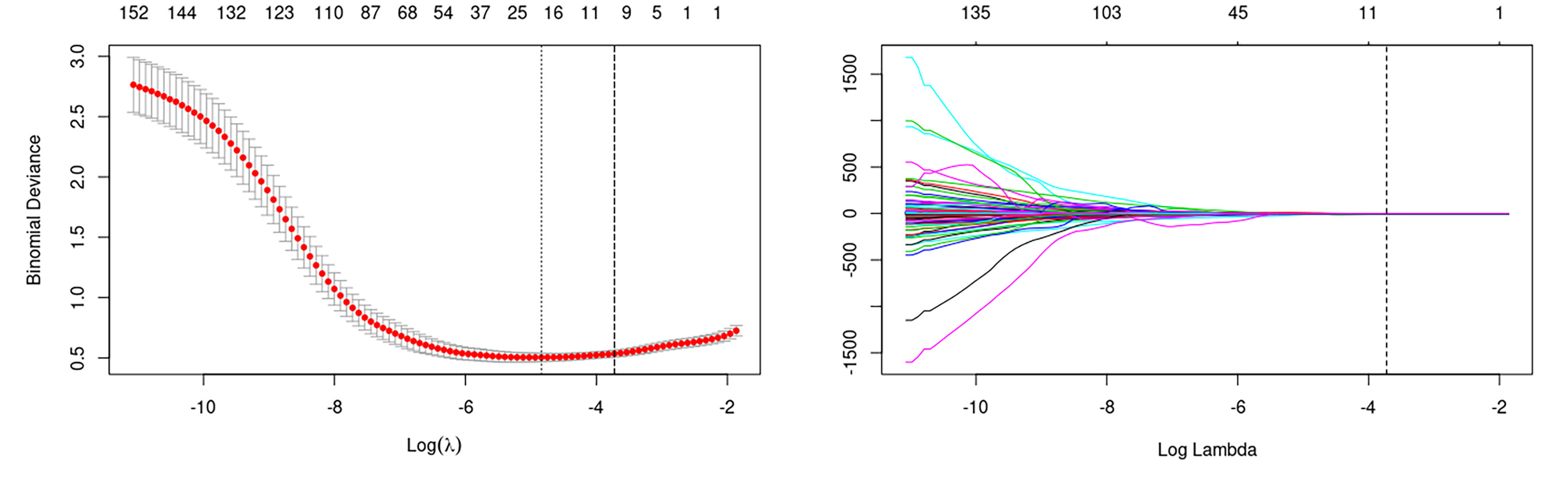

Supplement: Supplementary file 1 — Additional file 1: Supplementary Figure 1. Inter- and intra-observer Bland-Altman plots of measurement variability in 60 SSNs. SSNs=subsolid nodules, CI=confidence interval. Supplementary Figure 2. The process of select radiomics features. LASSO=least absolute shrinkage and selection operator. [file 12880_2023_1143_MOESM1_ESM.zip › Supplementary Figure 2.jpg]
